# Stage-specific meniscal features predict progression of osteoarthritis of the knee: a retrospective cohort study using data from the osteoarthritis initiative

**DOI:** 10.1186/s12891-019-2413-7

**Published:** 2019-01-22

**Authors:** Tsuneo Kawahara, Takahisa Sasho, Takashi Ohnishi, Hideaki Haneishi

**Affiliations:** 1 Graduate School of Engineering, Chiba University, 1-33 Yayoi-cho, Inage-ku, Chiba, 263-8522 Japan; 2 Medical Corporation Jinseikai, Chiba, Japan; 3 Graduate School of Medicine Research Institute Orthopedics, Chiba University, Chiba, Japan; 4 Center for Preventive Medicine, Chiba University, Chiba, Japan; 5 Center for Frontier Medical Engineering, Chiba University, Chiba, Japan

**Keywords:** Meniscal shape, Osteoarthritis of the knee, Osteoarthritis initiative (OAI), Shape measurement, MRI

## Abstract

**Background:**

In the progression of osteoarthritis (OA) of the knee, a correlation between meniscal posterior segment injuries and medial meniscal extrusion has been reported, but there have been few reports on the relationship with the meniscal shape. The purpose of this study was to clarify the features of the meniscal shape involved in the progression of knee OA.

**Methods:**

Data were obtained from the Osteoarthritis Initiative (OAI) database. We defined two sets of subjects. One set included 455 knees of subjects whose OA grade on the Kellgren Lawrence (KL) scale progressed in 24 months from baseline and the other set consisted of 455 knees with no progression. The OA progressed subjects were divided to three groups: the “OA change group”, KL0 and KL1 knees that progressed to KL2 and KL3; the “mild change group”, KL2 knees that progressed to KL3; and the “severe change group”, KL2 and KL3 knees that progressed to KL4. The no progression set was divided into three groups whose OA grade remained unchanged. We used magnetic resonance imaging data and manually measured seven items (longitudinal diameter [LD], anterior wedge thickness, anterior wedge width, posterior wedge width, posterior wedge thickness, anterior wedge angle, posterior wedge angle) from the sagittal slice and the extrusion from the coronal slice. These measurements were compared between knees with and without OA progression.

**Results:**

In the “OA change group” and “mild change group”, the anterior and posterior wedge widths and the extrusion were significantly larger, but the anterior and the posterior wedge angles were significantly smaller. In the “severe change group,” the LD and the extrusion were significantly larger. In each group, there was no uniform tendency for the correlation coefficient of the parameters evaluated.

**Conclusions:**

Our findings suggested (1) a larger meniscal LD at the baseline predicted progression of knee OA after 24 months and (2) a larger meniscal width and smaller meniscal angle predicted progression of knee OA after 24 months.

## Background

Osteoarthritis (OA) of the knee is well known as a multifactorial disease [1], comprising local and general factors. Among local factors, the role of the menisci in maintaining the local physiological mechanical environment is well established and their failure to fulfill this role leads to knee OA development or its progression. The meniscus is a fibrocartilage tissue present at the femoro-tibial joint that plays important roles in the knee including shock buffering and cartilage protection [2]. There have been many reports on the relationships between knee OA and the meniscus, and especially the relationships with medial meniscal extrusion have received considerable attention recently [3–12]. Some reports have addressed the relationship between meniscal extrusion and the presence of a meniscal posterior section injury [3–6]. There have been several studies reporting meniscal quantitative evaluation [7–12]. Among them, in two studies the severity of the knee OA was semi-quantitatively evaluated. The state of the meniscus was comprehensively assessed by Hunter et al. [7] using the Boston Leeds Osteoarthritis Knee Score (BLOKS), while Bloecker et al. [8] used Magnetic resonance imaging Osteoarthritis Knee Score (MOAKS). In addition, Wirth et al. showed that the joint space narrow (JSN) score was more severe and the amount of extrusion was larger [9]. In a different study, Bloecker et al. [10] divided the area of the femoral cartilage and tibial cartilage into eight segments and showed the correlation of cartilage loss in each segment with the amount of meniscal extrusion and the tibial plateau coverage. However, Emmanuel et al. [13] reported that tibial coverage was not beneficial in the quantitative assessment of meniscal extrusion and did not provide a common view on the quantitative parameters of the meniscus. Additionally, although the meniscal parameters such as width, thickness, and extrusion amount were described independently in previous reports [8–12], there have been no comprehensive studies on the relationship between each parameter and OA and the correlation between parameters for each OA grade.

We previously found a relationship between the severity of knee OA and the meniscal shape using magnetic resonance imaging (MRI) data of Japanese knees in a cross-sectional study but could not determine any relationship between meniscal shape and the longitudinal progression of knee OA [14]. There have been other longitudinal studies on knee OA [13–17], but there have been no reports on the involvement of the meniscal shape that predicts knee OA progression. The purpose of the present study was to clarify the features of the meniscal shape involved in knee OA progression.

## Methods

### Study participants

Image data were obtained from the Osteoarthritis Initiative (OAI) database. OAI is a multicenter database designed to identify risk factors associated with onset and progression of knee OA and to characterize disease biomarkers. Participants in the OAI were between 45 and 79 years of age at baseline and were of diverse races. Participants with rheumatoid arthritis or other inflammatory arthritis, with bilateral terminal knee OA (severe knee OA), those who were unable to walk without assistance, or with MRI contraindications were excluded. In addition, we excluded cases where the deformation of the meniscus was severe, and the quantitative measurement was difficult to obtain. Conversely, we did not exclude other meniscus injuries such as trauma, anterior cruciate ligament (ACL) injuries, etc. The OAI study was approved by the institutional review boards at each OAI clinical site and at the coordinating center (Memorial Hospital of Rhode Island Institutional Review Board, The Ohio State University’s Biomedical Sciences Institutional Review Board, University of Pittsburgh Institutional Review Board, University of Maryland Baltimore – Institutional Review Board, and Committee on Human Research at University of California, San Francisco). All participants provided written informed consent to the OAI study.

**Fig. 1:**
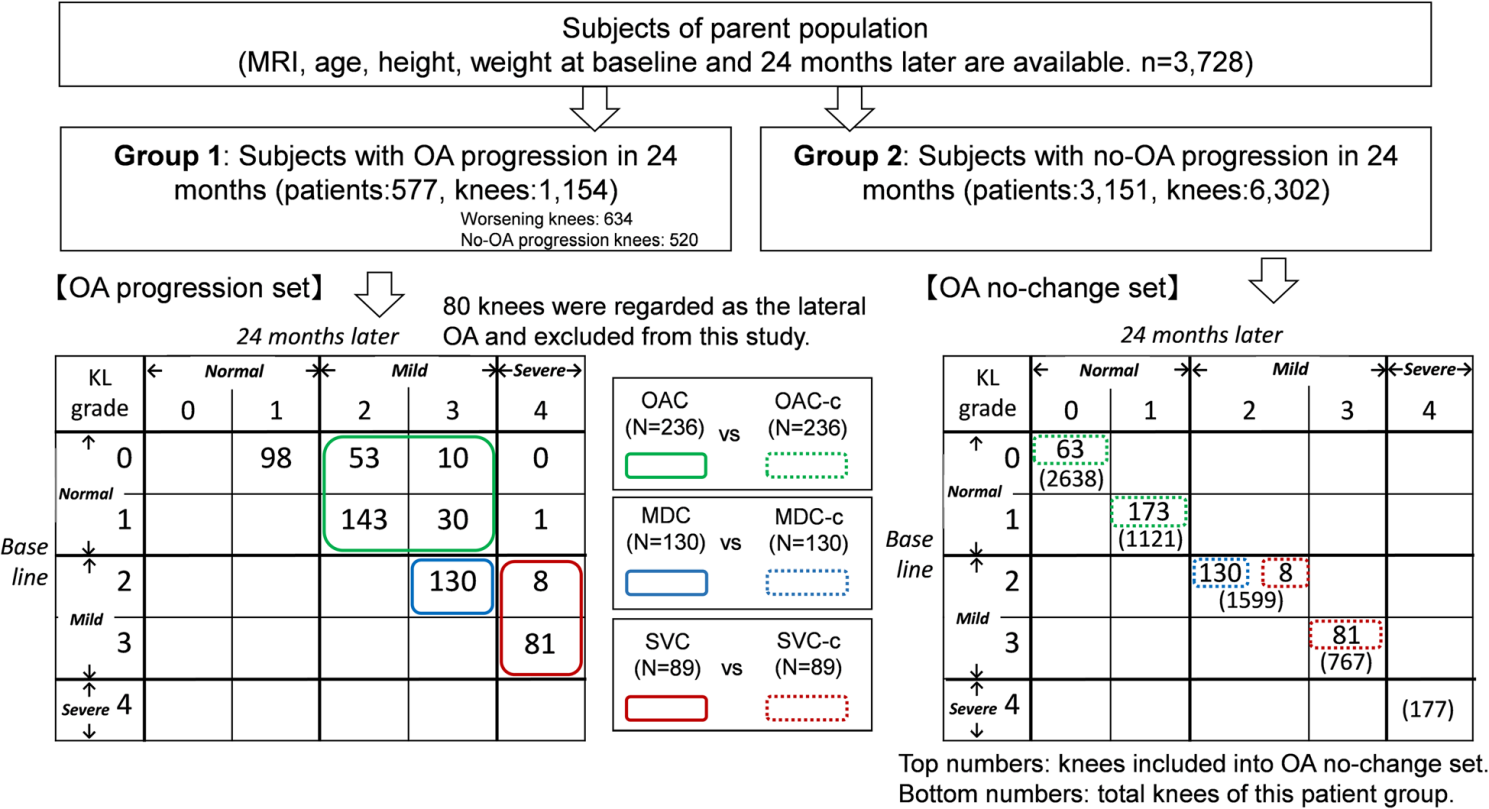
Selection flowchart of subjects and knees

The selection flowchart of the subjects and the knees is shown in Fig. 1. Initially, from the OAI database, 3728 subjects who had both MRI data and basic information such as age, height and weight at baseline and 24 months later were extracted. Each knee OA had been graded by the Kellgren Lawrence (KL) grade [18]. The KL grades were determined by two expert readers from the X-ray photography (XP). The severity classification by KL grade is used in most general clinics. In addition, we compared the severity classification by KL grade from the XP and a score denominated whole-organ magnetic resonance imaging score (WORMS) from MRI data used in our previous study and obtained results that were roughly consistent. Therefore, we assumed that adoption of the KL grade was reasonable [14].

The 3728 subjects were divided into two groups: Group 1 consisted of subjects with knee OA progression in one or both knees over 24 months, and Group 2 consisted of subjects without knee OA progression in both knees over 24 months. Worsen knee cases based on KL grade were extracted from Group 1 and we call those cases “OA progression set”. OA progression set contained 634 knees of the 577subjects. The control group was extracted from Group 2 by calling them as “OA no-change set”. OA no-change set contained 6302 knees of 3151 subjects. In OA progression set, cases of unilateral OA were excluded from this study because the mechanism of deformation of the meniscus was thought to be different from that of medial OA. The OAI database reports lateral joint space narrowing (XRJSL) and medial joint space narrowing (XRJSM) as the evaluation score of the joint space. We considered cases as lateral OA when the XRJSL value was larger than that of the XRJSM. The two tables at the bottom of Fig. 1 show the numbers of knees in each case of transition of KL grade in each group. In this report, we classified the severity of each knee OA into three classes as in our previous report [14]: we defined “normal” as KL0 and KL1 grades, “mild” as KL2 and KL3 grades, and “severe” as KL4.

We further defined three sub-types of OA progression for cases in the OA progression set. The first was the “OA change group” (OAC) in which KL0 or KL1 grades advanced to KL2 or KL3 grades and consisted of 236 knees. The second was the “mild change group” (MDC) in which KL2 advanced to KL3 and consisted of 130 knees. The third was the “severe change group” (SVC) in which KL2 or KL3 advanced to KL4 and consisted of 89 knees. The bottom left of Fig. 1 shows the detailed numbers of knees for each type.

For each of the three groups in the OA progression set, a control group was defined as the OA no-change set. As a control of the target “OA change group” (OAC), the same number of knees whose KL grade was KL0 or KL1 at baseline and did not change after 24 months were extracted. This group was named OAC-c. (Fig. 1, green dotted line). As a control of the target “mild change group” (MDC), the same number of knees whose KL grade remained constant at KL2 was extracted. This group was named MDC-c (Fig. 1, blue dotted line). Similarly, as a control of the “severe change group” (SVC), the same numbers of knees with KL2 or KL3 at baseline that did not change after 24 months were extracted. This group was defined SVC-c (Fig. 1, red dotted line). In contrast to the “OA change set,” knee selection from the parent population was arbitrary. Knees of the control group for each comparison were selected from the smallest ID numbers in Group 2 under two constraints. One was that Student’s t-test did not show any difference in average value of age (*p* < 0.05) and the other was that samples of KL2-to-KL2 knees selected as MDC-c and SVC-c showed no overlap.

In this report, we examined differences in the meniscal shape at baseline in three cases, between the OAC and OAC-c, between MDC and MDC-c, and between SVC and SVC-c.

### Magnetic resonance images and segmentation

Magnetic resonance images were taken using a 3 Tesla Magnetom Trio magnet (Siemens Erlangen, Germany), equipped with a quadrature transmitter and knee coil receiver. The OAI database contained images of some sequences, including a three-dimensional double echo steady state (3D_DESS), an intermediate weighted turbo spin echo (IW_TSE), and T2 mapping (T2_MAP; only on the right side) in the sagittal plane with an intermediate weighted turbo spin echo (IW_TSE), multiple section reconstruction (MPR), and T1 three-dimensional high speed low angle shooting (T1_3 D_FLASH [only on the right side]) in the coronal plane. However, in this study we used only images of the IW_TSE sequence to facilitate segmentation of the meniscus in either the sagittal or coronal plane. The slice thickness in the IW TSE was 3.0 mm and the resolution was 2.8 mm^2^/pixel.

In the OAI database, the knee positioning is defined in the “MRI Procedure Manual for Examinations of the Knee and Thigh” (accessible at: https://data-archive.nimh.nih.gov/oai/). There is no additional protocol for knee flexion contracture in the MRI protocol. The manual indicates that if the positioning is unsuitable, it is to be commented on by a technician. However, there were no data with a comment in the data used in this study.

**Fig. 2:**
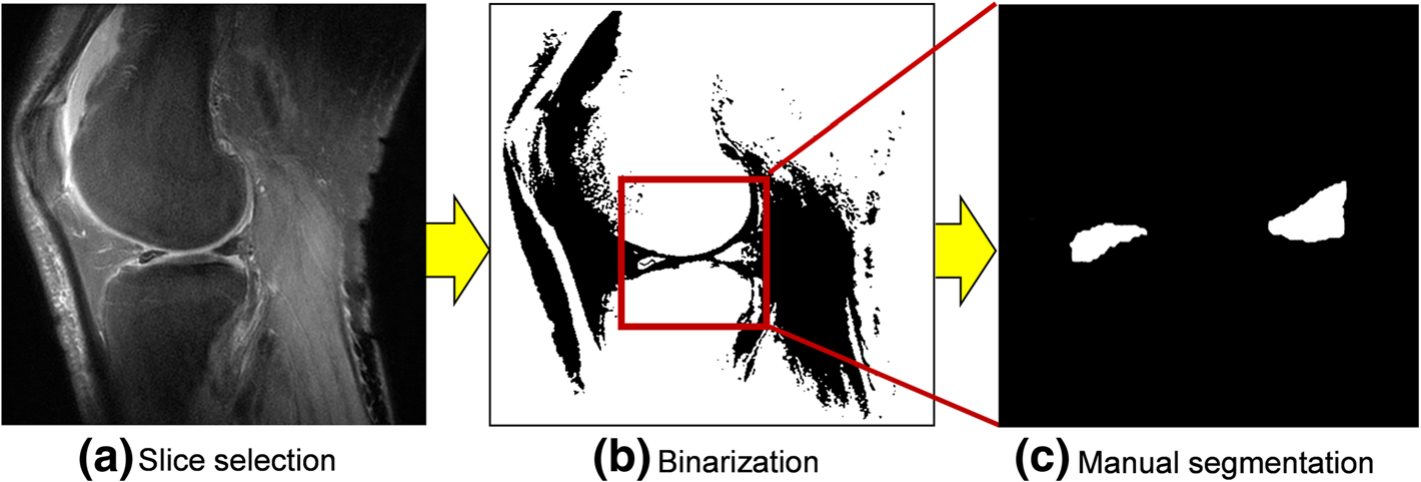
Meniscus segmentation procedure. **a** Selection of a slice with the maximum meniscal longitudinal diameter (LD). **b** Binarization of images with an appropriate threshold. **c** Extraction of the meniscal part of the binarized image by manual segmentation

In order to measure the shape of the meniscus, sagittal plane slices with the largest longitudinal diameter (LD) of the medial meniscus were chosen by measuring the LD of several sagittal slices manually. We drew straight parallel lines from the outside of the anterior section and posterior section of the meniscus and defined the distance between them as LD. In order to measure the amount of extrusion of the medial meniscus, a coronal plane slice was determined based on “mid-coronal” section indicated by Bruns et al. [19]. The slice presents the greatest area of the medial spine. The segmentation method of the meniscus was carried out as in our past study [14] as shown in Fig. 2.

### Quantitative measurements

**Fig. 3:**
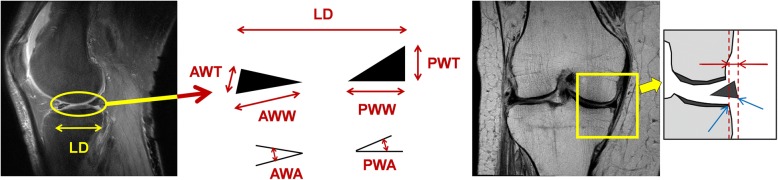
Quantitative measurements of the meniscus. Left: Geometric quantities for analysis of meniscal shape, LD (longitudinal diameter), AWT (anterior wedge thickness), AWW (anterior wedge width), PWW (posterior wedge width), PWT (posterior wedge thickness), AWA (anterior wedge angle), PWA (posterior wedge angle). Right: Amount of meniscal extrusion. The tibial end and meniscal end are manually identified from the magnetic resonance coronal slice (blue arrows), then the horizontal distance between them (red arrows) is defined as the amount of meniscal extrusion

The following quantities as shown in Fig. 3 (left) were measured using the sagittal slice:

- Longitudinal diameter (LD)
- Anterior wedge thickness (AWT)
- Anterior wedge width (AWW)
- Posterior wedge width (PWW)
- Posterior wedge thickness (PWT)
- Anterior wedge angle (AWA)
- Posterior wedge angle (PWA)

Measurements were obtained using the free image analysis software, ImageJ 1.47v (Wayne Rasband National Institutes of Health, USA). Each geometric quantity in the sagittal image of the meniscus was measured four times, the maximum value and the minimum value were excluded, and the average of the remaining two values was adopted. Measurements were taken by the author (KT) alone. Examiner reliability in meniscus shape measurement was examined using intraclass correlation coefficients (ICC), a one-way model proposed by McGraw & Wong [20]. In principle, ICC ranges from 0 to 1 and generally it is judged to be highly reliable with values over 0.7. For the measurements in this study, the ICC ranged 0.92 to 0.99, which confirmed its high reliability.

Each measurement value other than the angle was normalized by the height of the subject. Van Thiel et al. proposed an effective regression model that accurately predicted the required transplantation meniscal size using variables of height, weight, and sex, and reported a slightly more accurate result compared to previous reports [21]. In addition, they stated that the height of the patient is a good predictor of the meniscus size. Based on this report, standardization by patient height was considered appropriate [21]. The amount of extrusion was measured on the MR coronal image and was defined as the distance between the outer end of the tibia and the outer end of the meniscus (Fig. 3, right).

### Statistical analysis

For each of the three comparisons between the target and control groups, we compared the average values of confounding factors such as age, height, weight, and BMI and no significant differences were observed. In terms of the parameters regarding the size of each meniscus to be compared, a significant difference was shown by Student’s t-test and Welch’s t-test according to the situation of dispersion (*p* < 0.05). Correlations between parameters in each knee OA progression group and those in each control group were verified using Pearson’s correlation coefficient.

## Results

Table 1-a lists basic patient information, (i.e., age, height, weight, BMI) for subjects in the first verification. Table 2 shows raw data and standardized data of six groups: OAC, OAC-c, MDC, MDC-c, SVC and SVC-c. Although significant differences between XXX and XXX-c are shown with * or ** in Table 2, the differences only were summarized in Table 3.

**Table 1:** Basic information for the subjects in the three verifications (“a”, “b”, “c”)

	Age (y/o)	Height (cm)	Weight (kg)	BMI (kg/m2)
a. Basic information of OAC and OAC-c				
OAC (Normal→Mild)	59.4 ± 8.2	167.2 ± 8.4	82.0 ± 13.5	29.2 ± 4.0
OAC-c (Normal)	61.3 ± 8.8	167.8 ± 8.9	79.4 ± 16.2	28.2 ± 5.3
b. Basic information of MDC and MDC-c				
MDC (Mild(KL2) → Mild(KL3))	63.2 ± 8.1	166.1 ± 8.9	85.2 ± 17.8	30.7 ± 5.1
MDC-c (Mild(KL2))	65.6 ± 9.5	167.6 ± 8.4	80.6 ± 16.9	28.6 ± 5.1
c. Basic information of SVC and SVC-c				
SVC (Mild→Severe)	63.0 ± 7.8	167.8 ± 8.6	84.8 ± 14.7	30.0 ± 4.1
SVC-c (Mild)	64.3 ± 9.2	168.2 ± 8.0	83.9 ± 16.6	29.5 ± 4.7

**Table 2:** Measurement results of medial meniscal size and the amount of extrusion in the first verification

a-1) Raw data of OAC and OAC-c								(* *p* < 0.05, ** *p* < 0.01)
	LD(mm)	AWT(mm)	AWW(mm)	PWW(mm)	PWT(mm)	AWA(degree)	PWA(degree)	Extrusion(mm)
OAC (Normal→Mild)	44.21 ± 4.18	6.54 ± 1.15	10.11 ± 1.36	14.44 ± 2.33	6.43 ± 1.30	42.3 ± 7.4	28.0 ± 5.9	2.01 ± 0.81
OAC-c (Normal)	43.64 ± 3.63	6.22 ± 1.07	9.14 ± 1.35	13.06 ± 1.81	6.31 ± 1.15	44.1 ± 6.1	31.5 ± 6.8	1.46 ± 0.53
						*	**	**
a-2) Normalized data of OAC and OAC-c								
	LD(10-3 mm)	AWT(10-3 mm)	AWW(10-3 mm)	PWW(10-3 mm)	PWT(10-3 mm)	AWA(degree)	PWA(degree)	Extrusion(mm)
OAC (Normal→Mild)	26.42 ± 1.93	3.91 ± 0.66	6.50 ± 0.79	8.64 ± 1.31	3.84 ± 0.75	42.3 ± 7.4	28.0 ± 5.9	2.01 ± 0.81
OAC-c (Normal)	26.06 ± 1.61	3.67 ± 0.60	5.52 ± 0.84	7.75 ± 1.00	3.85 ± 0.64	44.1 ± 6.1	31.5 ± 6.8	1.46 ± 0.53
		**	**	**		*	**	**
b-1) Raw data of MDC and MDC-c								
	LD(mm)	AWT(mm)	AWW(mm)	PWW(mm)	PWT(mm)	AWA(degree)	PWA(degree)	Extrusion(mm)
MDC (Mild(KL2) → Mild(KL3))	44.78 ± 4.06	6.76 ± 1.44	10.28 ± 1.71	13.24 ± 1.85	6.67 ± 1.16	43.3 ± 7.5	31.9 ± 6.6	3.07 ± 0.99
MDC-c (Mild(KL2))	45.40 ± 3.75	6.79 ± 1.11	9.78 ± 1.37	11.75 ± 2.06	6.66 ± 1.21	48.2 ± 9.3	38.8 ± 8.8	2.77 ± 0.85
			*	**		**	**	*
b-2) Normalized data of MDC and MDC-c								
	LD(10-3 mm)	AWT(10-3 mm)	AWW(10-3 mm)	PWW(10-3 mm)	PWT(10-3 mm)	AWA(degree)	PWA(degree)	Extrusion(mm)
MDC (Mild(KL2) → Mild(KL3))	26.95 ± 1.79	4.07 ± 0.84	6.19 ± 0.99	7.97 ± 1.02	4.02 ± 0.67	43.3 ± 7.5	31.9 ± 6.6	3.07 ± 0.99
MDC-c (Mild(KL2))	27.09 ± 1.73	4.05 ± 0.66	5.84 ± 0.76	7.02 ± 1.22	3.98 ± 0.75	48.2 ± 9.3	38.8 ± 8.8	2.77 ± 0.85
				*		**	**	*
c-1) Raw data of SVC and SVC-c								
	LD(mm)	AWT(mm)	AWW(mm)	PWW(mm)	PWT(mm)	AWA(degree)	PWA(degree)	Extrusion(mm)
SVC (Mild→Severe)	47.87 ± 4.42	6.96 ± 1.14	10.04 ± 1.57	11.31 ± 2.14	6.88 ± 1.32	47.5 ± 8.9	39.9 ± 9.2	3.56 ± 0.99
SVC-c (Mild)	45.32 ± 3.82	6.70 ± 1.14	9.80 ± 1.30	11.51 ± 2.10	6.49 ± 1.19	47.6 ± 9.3	39.2 ± 9.8	2.87 ± 0.95
	**							**
c-2) Normalized data of SVC and SVC-c								
	LD(10-3 mm)	AWT(10-3 mm)	AWW(10-3 mm)	PWW(10-3 mm)	PWT(10-3 mm)	AWA(degree)	PWA(degree)	Extrusion(mm)
SVC (Mild→Severe)	28.53 ± 2.10	4.15 ± 0.66	5.98 ± 0.88	6.74 ± 1.25	4.10 ± 0.77	47.5 ± 8.9	39.9 ± 9.2	3.56 ± 0.99
SVC-c (Mild)	26.93 ± 1.75	3.99 ± 0.67	5.83 ± 0.74	6.84 ± 1.22	3.86 ± 0.70	47.6 ± 9.3	39.2 ± 9.8	2.87 ± 0.95
	**							**

This table shows the mean ± standard deviation. “a-1”, “b-1”, “c-1” lists the measured values (raw data) and “a-2”, “b-2”, “c-2” lists the values normalized by the height of each subject. Significant differences between the two groups are indicated by one or two asterisks (*p < 0.05, **p < 0.01)

**Table 3:** Statistical significant differences in measurement parameter of the meniscus

a-1) Raw data of OAC and OAC-c								(* *p* < 0.05、** *p* < 0.01)
	LD(mm)	AWT(mm)	AWW(mm)	PWW(mm)	PWT(mm)	AWA(degree)	PWA(degree)	Extrusion(mm)
OAC (Normal→Mild)						*	**	**
a-2) Normalized data of OAC and OAC-c								
	LD(10-3 mm)	AWT(10-3 mm)	AWW(10-3 mm)	PWW(10-3 mm)	PWT(10-3 mm)	AWA(degree)	PWA(degree)	Extrusion(mm)
OAC (Normal→Mild)		**	**	**		*	**	**
b-1) Raw data of MDC and MDC-c								
	LD(mm)	AWT(mm)	AWW(mm)	PWW(mm)	PWT(mm)	AWA(degree)	PWA(degree)	Extrusion(mm)
MDC (Mild(KL2) → Mild(KL3))			*	**		**	**	*
b-2) Normalized data of MDC and MDC-c								
	LD(10-3 mm)	AWT(10-3 mm)	AWW(10-3 mm)	PWW(10-3 mm)	PWT(10-3 mm)	AWA(degree)	PWA(degree)	Extrusion(mm)
MDC (Mild(KL2) → Mild(KL3))				*		**	**	*
c-1) Raw data of SVC and SVC-c								
	LD(mm)	AWT(mm)	AWW(mm)	PWW(mm)	PWT(mm)	AWA(degree)	PWA(degree)	Extrusion(mm)
SVC (Mild→Severe)	**							**
c-2) Normalized data of SVC and SVC-c								
	LD(10-3 mm)	AWT(10-3 mm)	AWW(10-3 mm)	PWW(10-3 mm)	PWT(10-3 mm)	AWA(degree)	PWA(degree)	Extrusion(mm)
SVC (Mild→Severe)	**							**

We extracted the points where there was a statistically significant difference in the meniscal measurement parameters in each verification

Significant differences between the two groups are indicated by one or two asterisks (*p <0.05, **p<0.01)

Table 2 a-1) and Table 2 a-2) report the meniscal shapes at baseline of the OAC and OAC-c groups. There was a significant difference in AWA: 42.3 ± 7.4 vs 44.1 ± 6.1 degree, PWA: 28.0 ± 5.9 vs 31.5 ± 6.8 degree, and extrusion: 2.01 ± 0.81 vs 1.46 ± 0.53 mm. For data normalized by the height of each subject, there was a significant difference between the two groups in AWT: 3.91 ± 0.66 vs 3.67 ± 0.60 10^− 3^ mm, AWW: 6.50 ± 0.79 vs 5.52 ± 0.84 10^− 3^ mm, PWW: 8.64 ± 1.31 vs 7.75 ± 1.00 10^− 3^ mm, AWA: 42.3 ± 7.4 vs 44.1 ± 6.1 degree, PWA: 28.0 ± 5.9 vs 31.5 ± 6.8 degree, and extrusion: 2.01 ± 0.81 vs 1.46 ± 0.53 mm.

Table 1-b lists the basic information for subjects in the second verification. Table 2 b-1) and Table 2-b-2) reports the meniscal shapes at baseline of the MDC and MDC-c groups. There was a significant difference between the two groups in AWW: 10.28 ± 1.71 vs 9.78 ± 1.37 mm, PWW: 13.24 ± 1.85 vs 11.75 ± 2.06 mm, AWA: 43.3 ± 7.5 vs 48.2 ± 9.3 degree, PWA: 31.9 ± 6.6 vs 38.8 ± 8.8 degree, and extrusion: 3.07 ± 0.99 vs 2.77 ± 0.85 mm. For the data normalized by height of each subject, there was a significant difference between the two groups in PWW: 7.97 ± 1.02 vs 7.02 ± 1.22 10^− 3^ mm, AWA: 43.3 ± 7.5 vs 48.2 ± 9.3 degree, PWA: 31.9 ± 6.6 vs 38.8 ± 8.8 degree, and extrusion: 3.07 ± 0.99 vs 2.77 ± 0.85 mm.

Table 1-c lists basic information of the subjects in the third verification. Table 2 c-1) and Table 2-c-2) reports the meniscal shapes at baseline of the SVC and SVC-c groups. There was a significant difference between the two groups in LD: 47.87 ± 4.42 vs 45.32 ± 3.82 mm and extrusion: 3.56 ± 0.99 vs 2.87 ± 0.95 mm. For the data normalized by the height of each subject, there was a significant difference between the two groups in LD: 28.53 ± 2.10 vs 26.93 ± 1.75 10^− 3^ mm and extrusion: 3.56 ± 0.99 vs 2.87 ± 0.95 mm.

Table 4 shows the correlation between basic information and measurement parameters of each group for the three verifications. “a” in Table 4 shows the correlation coefficients between the parameters of the each knee OA progression group: 1) OAC, 2) MDC, 3) SVC. “b” shows those of control groups 1) OAC-c, 2) MDC-c, 3) SVC-c for “a”. Commonly, for each group, weight and BMI showed a high correlation (*r* = 0.79–0.87), while height and weight showed a moderate correlation (*r* = 0.45–0.59). The parameters of the meniscus were not correlated with any of the basic data. For all groups except for SVC-c, the LD and PWT showed a moderate positive correlation (*r* = 0.40–0.52) and the PWW and PWA showed a moderate inverse correlation (*r* = − 0.56–-0.40). Combination of parameters showing other moderate or higher correlation coefficients were not constant. There was also no specific tendency in only the groups in which the knee OA progressed or only in the group in which the knee OA did not progressed.

**Table 4:**
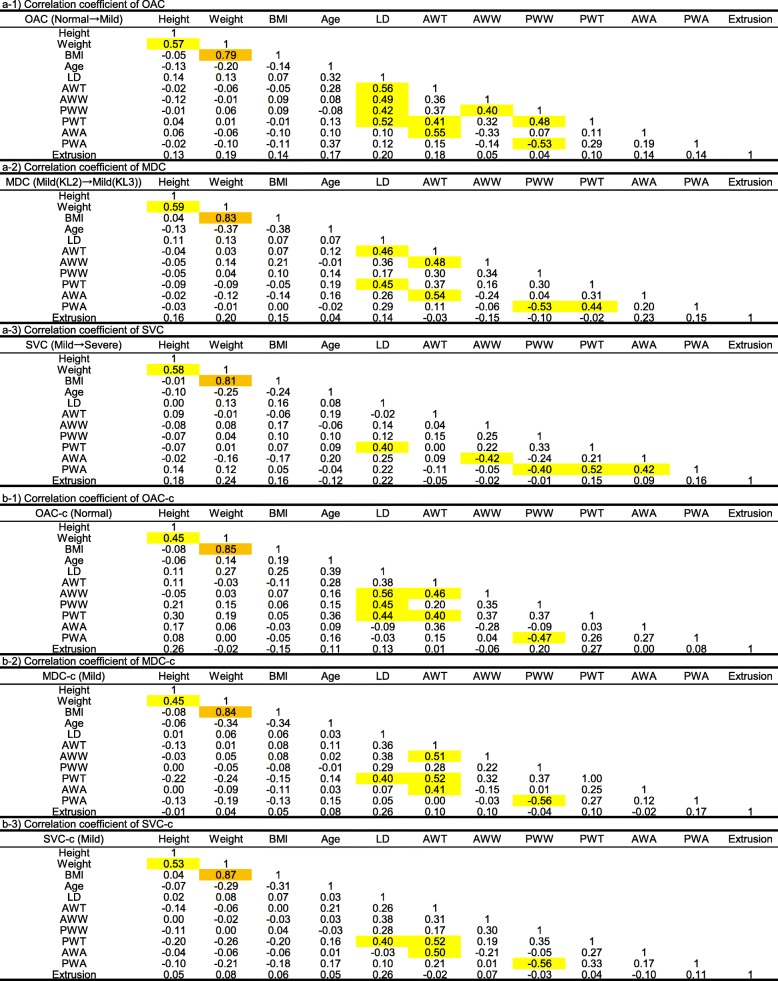
Correlation of measurement parameters in each group

The correlation coefficient between the basic information and measurement parameter is shown. The orange cells have a high correlation of 0.6 or more, and the yellow cells have a moderate correlation of 0.4 or more

## Discussion

This study measured the shape of the meniscus at baseline for all groups, i.e. OAC, MDC, and SVC with knee OA progression and the control groups without knee OA progression after 24 months. As a result, the meniscal shape prior to knee OA progression differed in terms of OAC, MDC, and SVC features, respectively. For the OAC and the MDC, the meniscal angles were significantly larger. For the SVC, the LD was significantly larger. The amount of extrusion was found to be significantly larger in the all groups with knee OA progression. The characteristics of each parameter are discussed below.

### Longitudinal study period

There have been several reports on the relationship between the progression of knee OA and meniscal shape [13–17]. The meniscus showed minor damage without any specific shape changes in early OA, and then medial extrusion was observed accompanied by degeneration or damage in the posterior region. However, it was not clear whether the OA progression occurred before or after the changes in meniscal shape. Several longitudinal studies about progression of knee OA and joint structure have been reported. Emmanuel et al. [13] carried out longitudinal tracking for four years and found that early meniscal extrusion was associated with knee OA progression. Roemer et al. [17] showed that certain structural features of joint damage appeared on MRI two years before the onset of knee OA, and those knees for which the above-mentioned features were newly recognized showed a higher risk of knee OA. From these results, we considered that the period of 24 months adopted in this study was reasonable as the shortest time to observe differences in OA changes in subjects.

### Meniscal size change

In this study, parameters with the length dimension were normalized, while parameters associated with angular dimension were not. In Table 2, comparison of extrusion in a), b) and c) and comparison of LD in c) show the same significant difference in both raw and normalized data. In terms of AWT, AWW and PWW in a), only normalized data showed the difference. In terms of AWW and PWW in b), raw data showed higher significant differences than the normalized data. In all verifications, the parameters of meniscal angle, extrusion and LD seem be stable and useful parameters for predicting the condition after 24 months.

**Fig. 4:**
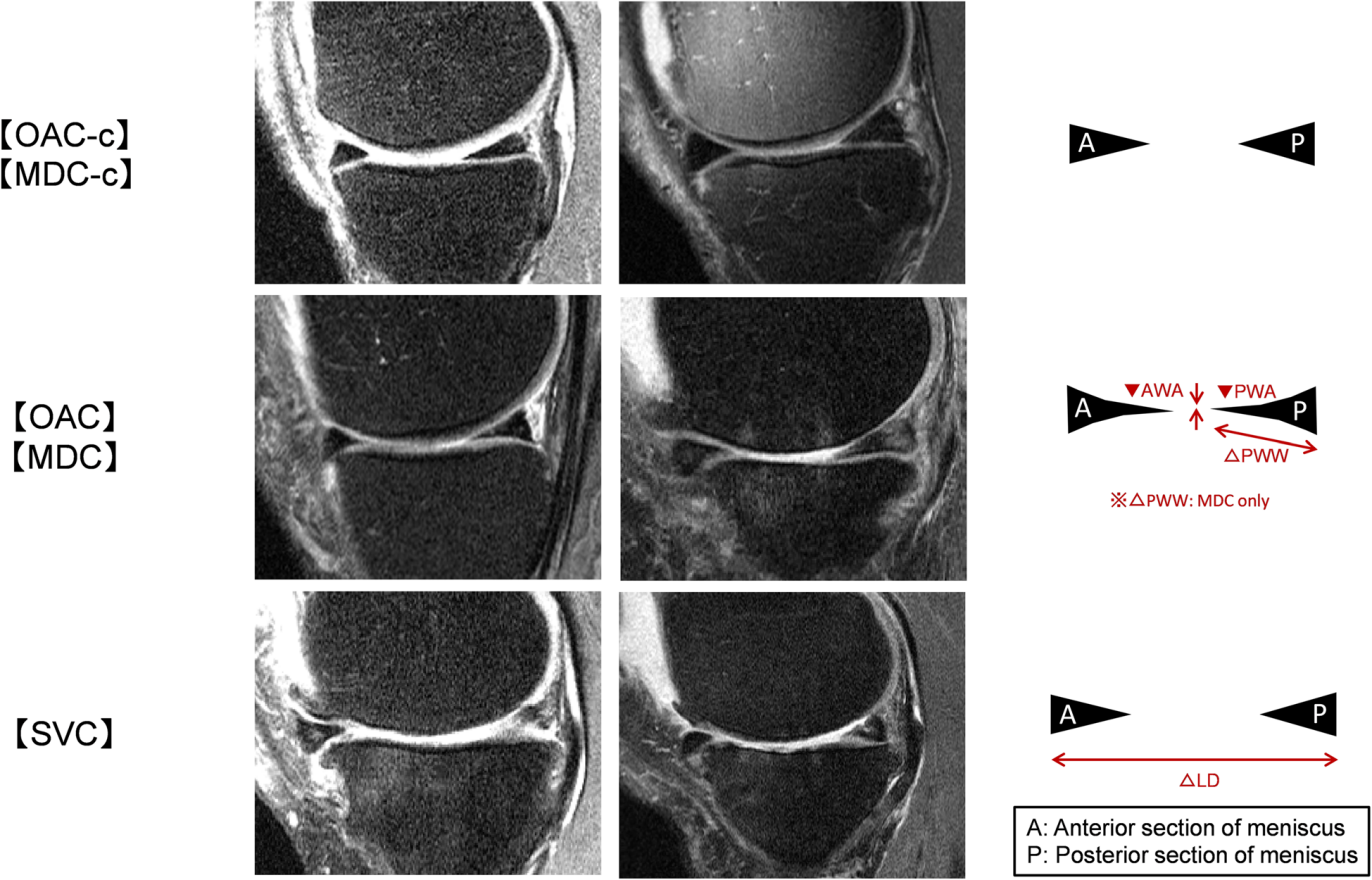
Features of meniscal shape in each group. Two example magnetic resonance images of each group are shown. Schematic diagram of meniscal change is shown on the right side. “A” indicates the anterior section of the meniscus and “P” indicates the posterior section of the meniscus. Red letters indicate changes in parameters, Δ indicates that it is larger than normal, and ▼ indicates that it is smaller than normal

Examples of results of the meniscal shape change obtained are shown in Fig. 4. From these results, we could suggest that knee OA became more severe when an enlargement of the meniscal LD occurred, because it is recognized that a statistically significant difference of LD is a feature of the meniscus shape. Regarding knee OA progression and LD enlargement, we observed a strong correlation in our previous study [14]; however, the preliminary LD enlargement was considered to be one element of knee OA severity from the present study. Enlargement of the LD was thought to cause the meniscus to deviate from the femoro-tibial joint in the anteroposterior direction, and the contact pressure of the femoro-tibial cartilage became stronger, and the OA became more severe.

In addition, we believed that it was possible to predict the progression of knee OA when the anterior/posterior meniscal angle decreased without any changes in the meniscal LD, because we found that there were statistically significant differences in meniscus angles in OAC vs OAC-c and MDC vs MDC-c, although there was no significant difference in LD. We considered that the meniscus in the normal case or the mild OA case had a small amount of extrusion and the LD did not increase, thus it could retain its original position while maintaining the hoop function. Therefore, we considered that the meniscus played a role in shock absorption of the femoro-tibial joint [2], and as shown in Fig. 4, the curvature of the upper and lower sides may be recognized in many cases based on the convex shape of the femoral cartilage and the tibial cartilage. However, if this crushed state continued, the impact absorption function of the meniscus might decrease, and potentially cause contact of the femoro-tibial cartilage, suggesting that it may lead to OA progression [22].

### Meniscal extrusion

For all verifications, we observed that the knee OA progressed as the amount of extrusion increased. Meniscal extrusion has been considered an important risk factor of knee OA progression in many previous studies and the results of the present study also provide further support [13–17].

### Correlation coefficient

Body weight and BMI, and height and weight are known to be strongly correlated. Knee OA shows significant degeneration in the posterior section of the medial meniscus, and the present study found a correlation between LD and PWT [3–6], while PWW and PWA showed a moderate correlation. We considered that the meniscus in the normal case or the mild OA case had a small amount of extrusion and the LD did not increase; thus, it could exist in its original position while maintaining the hoop function. Therefore, we considered the moderate correlation between PWW and PWA to be reasonable as the meniscus fulfills the shock absorbing function.

The meniscal extrusion mentioned above did not show any correlation with any of the parameters evaluated. Meniscal extrusion appeared to be an important risk factor of knee OA and it was an event occurring independently of meniscal shape changes.

### Limitations

There are some limitations to the present study. First, the sequences for MRI acquisition were limited. Since OAI data were used, sequences such as fast imaging with steady-state precession (FISP) and T2*, which have been used for segmentation of the meniscus in recent years, were not included [23]. Therefore, there may be a limitation in the detailed evaluation due to data loss. In addition, although the position of the meniscus varies depending on the load, this report used imaging data in the unloaded supine position. Second, this report only dealt with the medial meniscus and the lateral meniscus was not evaluated. We focused on the medial meniscus as only the medial meniscus showed significant changes in past reports [14]. Third, the adjustment for confounding factors was insufficient. The presence or absence of meniscal damage and malalignment affect the condition of the knee [24], but these were not considered in this study. Fourth, we did not follow up the actual meniscal shape after 24 months. Thus, in some cases the meniscus might have been injured in the period from the baseline and 24 months later and caused the progression of knee OA. In this paper, we could not measure the meniscal shape at after 24 months and distinguish when was the meniscus injured. A difference in OA progression by the meniscus injury will be investigated in future. Fifth, since it takes much time and effort to process data for each subject knee, a multilateral analysis using many subjects would be considered to be too difficult to establish.

## Conclusion

This paper is the first report to describe the features of the meniscal shape preceding the longitudinal progression of knee OA after 24 months. Our findings suggest that knee OA becomes severe after 24 months if enlargement of the meniscal LD is observed at baseline. In addition, even if the LD of the meniscus did not change, the fact that the meniscal width was large and the meniscal angle was small also suggested its potential role in the progress of the knee OA after 24 months.

In order to predict the progression of longitudinal knee OA, we simply compared geometrical parameters of the meniscus in the baseline with the “OA progression set” and the “OA no-change set” and identified the features of meniscal shape that had an impact on OA progression. Although we calculated the correlation coefficient between the parameters, we did not perform multiple regression analysis to determine to what degree each parameter affected knee OA progression. This analysis will be addressed in our future studies.

## Abbreviations

3D_DESS: Three-dimensional double echo steady state
ACL: Anterior cruciate ligament
AWA: Anterior wedge angle
AWT: Anterior wedge thickness
AWW: Anterior wedge width
BLOKS: Boston Leeds Osteoarthritis Knee Score
BMI: Body mass index
FISP: fast imaging with steady-state precession
IW_TSE: Intermediate weighted turbo spin echo
JSN: Joint space narrow
KL: Kellgren Lawrence
LD: Longitudinal diameter
MDC: Mild change group
MOAKS: MRI Osteoarthritis Knee Score
MPR: Multiple section reconstruction
MRI: Magnetic resonance imaging
OA: Osteoarthritis
OAC: OA change group
OAI: Osteoarthritis Initiative
PWA: Posterior wedge angle
PWT: Posterior wedge thickness
PWW: Posterior wedge width
SVC: Severe change group
T2_MAP: T2 mapping
WORMS: Whole-organ magnetic resonance imaging score
XP: X-ray photography
XRJSL: X-ray joint space lateral
XRJSM: X-ray joint space medial
